# A Case of a Father and Son With Complex Regional Pain Syndrome Type 1 Exhibiting Different Resting-State Functional Connectivity on Functional MRI

**DOI:** 10.7759/cureus.52589

**Published:** 2024-01-19

**Authors:** Katsuyuki Moriwaki, Atsuo Yoshino, Yumi Ikejiri, Ryuji Nakamura, Yasuo Tsutsumi

**Affiliations:** 1 Department of Anesthesiology, Hiroshima University Hospital, Hiroshima, JPN; 2 Department of Anesthesiology, Hiroshima Hiramatsu Hospital, Hiroshima, JPN; 3 Health Service Center, Hiroshima University, Higashi-Hiroshima, JPN; 4 Center for Brain, Mind and Kansei Sciences Research, Hiroshima University, Hiroshima, JPN; 5 Department of Anesthesiology, Shimura Hospital, Hiroshima, JPN

**Keywords:** phenotype, brain network, functional mri, resting-state functional connectivity, complex regional pain syndrome type 1

## Abstract

Complex regional pain syndrome (CRPS) type 1 is a chronic pain condition whose pathogenesis involves changes in the central and peripheral nervous systems, with potential genetic contributions. Functional magnetic resonance imaging (fMRI) studies report that alterations in resting-state functional connectivity (rsFC) may reflect central nervous system anomalies in CRPS type 1. Herein, we describe the case of a father and son with CRPS type 1 who exhibited different rsFC patterns in fMRI analyses correlating with their individual CRPS phenotypes. A 39-year-old male and his 61-year-old father presented with severe pain and mobility limitations in their right upper limbs following a vehicle accident and a fall, respectively, and were diagnosed with CRPS type 1. Despite receiving treatment, they experienced severe pain and limited mobility. The son exhibited dystonia and musculoskeletal atrophy while the father experienced extensive sensory disturbances. Bone scintigraphy revealed increased uptake in affected regions. The patients' resting-state fMRI data were compared with those of 48 healthy adults using the CONN software, with the false discovery rate set at p<0.05. Distinct brain regions for the father and son exhibited decreased rsFC (between the rostral prefrontal cortex and orbitofrontal cortex in the father and between the supplementary motor area and pallidum in the son; all in the right hemisphere). These changes corresponded to pain sensation and cognitive-emotional alterations in the father and limb movement disorders (dystonia) in the son. Our findings strongly support the idea that abnormalities in rsFC are closely linked to CRPS type 1 phenotypes.

## Introduction

Complex regional pain syndrome (CRPS) is a chronic pain syndrome that develops following fractures, soft tissue damage, or surgery and is characterized by pain, swelling, limited range of joint motion, vasomotor instability, skin changes, and patchy osteoporosis [1,2]. Although the pathogenesis is uncertain, classic and neurogenic inflammation in the affected limb's peripheral tissues and central nervous system pathophysiology, such as changes in nociceptive perception, contribute to its occurrence [1,2] Resting-state functional connectivity (rsFC) changes observed on functional magnetic resonance imaging (fMRI) have been recently investigated as indicators of central nervous system abnormalities in patients with CRPS [3-11]. Advanced research has identified various rsFC anomalies that are associated with alterations in pain perception and cognitive processes [3-9,11]. Some of these anomalies are also related to motor function disturbances [3,4,10]. Genetic contributions, including possible links between human leukocyte antigen (HLA) types and inflammatory responses related to CRPS, have also been suggested [1,2]. Recent studies have identified several genetic variants including inflammation-related genes in patients with CRPS [12,13]. Herein, we describe the case of a father and son with CRPS type 1 who, despite their close genetic relationship, exhibited different rsFC patterns on fMRI analyses, correlating with their individual CRPS phenotypes. This article was previously presented as a meeting abstract at the 2023 Japan Society of Pain Clinicians 57th Annual Meeting on July 15, 2023.

## Case presentation

This study was approved by our institutional review board for research ethics, and written informed consent was obtained from the patients before the study.

Patients

A 39-year-old male (height 160 cm, weight 50 kg) with no significant medical history had experienced a whiplash injury to the neck and contusions to the right arm and chest due to a motor vehicle accident. Initial management at a local hospital’s orthopedic department included various oral medications for pain relief and rehabilitation therapy. However, despite these interventions, the patient reported worsening spontaneous pain and progressive mobility limitations in the right upper limb. One year after the injury, the patient was referred to our pain clinic on suspicion of CRPS type 1.

The 61-year-old father (height 160 cm, weight 50 kg) with no significant medical history sustained a severe contusion to his right shoulder after falling down the stairs at home one year following his son’s accident. Diagnosed with acromioclavicular joint injury and treated at a local hospital’s orthopedic department, he experienced persistent and severe pain, along with a restricted range of motion in the right upper limb. He was referred to our pain clinic on suspicion of CRPS type 1 nine months after the initial injury.

**Figure 1:**
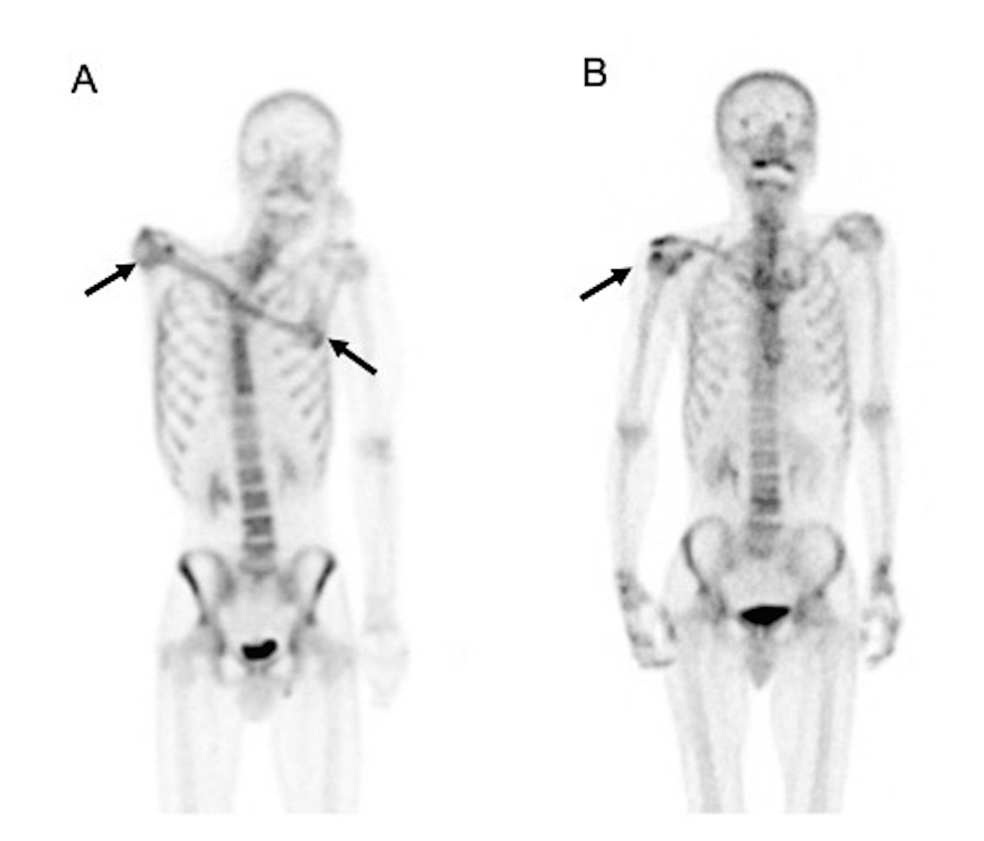
Bone scintigraphy findings in the (A) son and the (B) father

The father and son met the Budapest Criteria for CRPS and were diagnosed with CRPS type 1. Table 1 presents the symptoms, signs, treatments, and examination findings. The son presented with pronounced spontaneous pain, allodynia, dystonia, a refractory ulcer on the affected elbow, muscle atrophy, and joint contracture, accompanied by progressive musculoskeletal atrophy. However, the father did not exhibit progressive musculoskeletal atrophy in the affected limb. Instead, he displayed widespread sensory disturbances, including tactile hypoesthesia and allodynia spreading to the head, neck, and lower limb on the ipsilateral affected limb, neglect-like symptoms, and pain upon movement of the affected limb. Bone scintigraphy revealed increased technetium uptake in the peri-articular bones of the affected limb’s shoulder in the father and son and the elbow in the son (Figure 1). Both patients were prescribed a combination of medications, including a gabapentinoid (pregabalin), a serotonin-noradrenaline reuptake inhibitor (duloxetine), a weak opioid (tramadol), acetaminophen, a non-steroidal anti-inflammatory drug (loxoprofen), an anxiolytic (alprazolam), and a hypnotic orexin receptor antagonist (either lemborexant or suvorexant). These medications were consistently administered throughout our treatment regimen, with specific dosages meticulously tailored to meet the individual needs of each patient. Furthermore, the patients underwent a comprehensive treatment approach that included interventional, rehabilitation, and psychiatric therapies, as detailed in Table 1. However, both patients experienced limited pain relief. A resting-state fMRI was performed three years and five months after the accident for the son and two years and seven months after the injury for the father.

**Table 1:** The symptoms, signs, treatments, and examination findings of CRPS type 1 in the patients CRPS: complex regional pain syndrome; SNRI: serotonin and norepinephrine reuptake inhibitor; NSAID: non-steroidal anti-inflammatory drug; ROM: range of motion

Item	Son	Father
Course		
Inciting event	Automobile rear-end collision with cervical sprain, contusions to right back, anterior thorax, and forearm	Fall, right shoulder contusion, shoulder joint ligament damage
Age at injury (year old)	39	61
Period from injury to referral (months)	12	9
Symptoms and signs after consultation		
Pain and sensory	Motion-induced pain of the affected limb, hyperesthesia/hyperalgesia, allodynia (+++)	Motion-induced pain of the affected limb, hyperesthesia/hyperalgesia, allodynia (++), ipsilateral hemisphere tactile hypoesthesia, neglect-like symptoms (+)
Vasomotor	Temperature asymmetry (++), skin color changes, and asymmetry (++)	Temperature asymmetry (+), skin color changes, and asymmetry (+)
Sudomotor/edema	Sweating asymmetry (+), edema (+++)	Sweating asymmetry (+), edema (+/-)
Motor/trophic	Decreased ROM (+++), motor dysfunction: dystonia (+++), trophic changes: muscular atrophy, ulcer of the elbow	Decreased ROM (+++), motor dysfunction: dystonia (-), trophic changes (+/-)
Special Examination		
Thermography	Elevation of right upper limb skin temperature	Elevation of right-hand skin temperature
Bone scintigraphy (Tc99m)	Enhanced uptake around the right shoulder joint, humerus, and elbow joint (Figure 1A)	Increased uptake around the right shoulder joint, right-hand joint, and finger joints (compared to left) (Figure 1B)
Resting-state functional connectivity	Decreased functional connectivity between the supplementary motor area and the pallidum (Figure 2A)	Decreased functional connectivity between the rostral prefrontal cortex and the subgenual anterior cingulate cortex (Figure 2B)
Treatment		
Oral medication	Gabapentinoids, SNRI, weak opioids, acetaminophen, NSAIDs, anxiolytics, sleep medications	Gabapentinoids, SNRI, weak opioids, acetaminophen, anxiolytics, sleep medications
Interventional treatment	Trigger point injections (for contralateral secondary myofascial pain)	No interventional procedure (nerve blocks worsened the pain)
Psychiatric treatment	Supportive psychotherapy	Supportive psychotherapy
Rehabilitation Treatment	Exercise therapy not possible	Exercise therapy not possible

rsFC analysis

**Figure 2:**
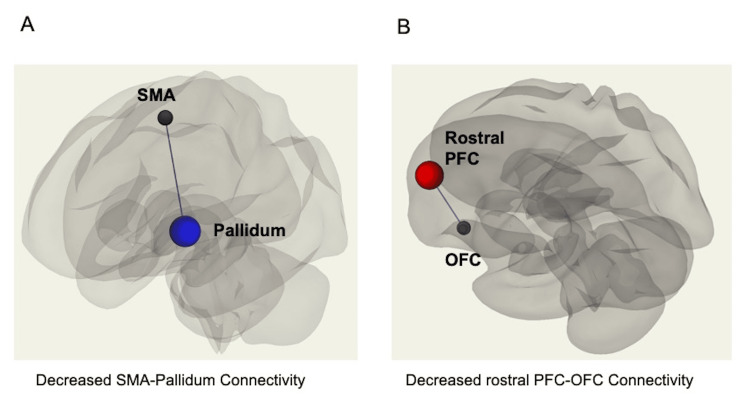
Abnormalities in brain functional connectivity during resting-state functional magnetic resonance imaging (fMRI) (A) Son's connectivity pattern: In the son, a decrease in functional connectivity was observed between the supplementary motor area (SMA) and the pallidum. (B) Father's connectivity pattern: In the father, a decrease in functional connectivity was noted between the rostral prefrontal cortex (PFC) and the orbitofrontal cortex (OFC).

Resting-state fMRI data were analyzed using SPM12 (Wellcome Department of Cognitive Neurology, London, UK), CONN [14], and MATLAB version 8.5 (R2017b, MathWorks, Natick, MA, USA). Region of interest (ROI)-to-ROI analyses were conducted for all ROIs defined within CONN. Resting-state fMRI data from the father and son with CRPS type 1 were compared with those of 48 healthy controls to identify variations in rsFC. The rsFC was analyzed using false discovery rate control, with the threshold set at p < 0.05. A decrease in rsFC between the rostral prefrontal cortex (PFC) and orbitofrontal cortex (OFC) was observed in the father. In contrast, a decrease in rsFC between the supplementary motor area (SMA) and the pallidum was observed in the son (Figure 2). These changes in rsFC were detected in the right hemisphere, corresponding to the side of the affected limb. Neither the father nor the son showed differences in the volume of each brain region within the anatomical ROI compared with that of healthy individuals.

## Discussion

CRPS phenotypes and rsFC

In our parent-offspring case of CRPS type 1, we observed decreased functional connectivity in different brain regions during resting-state fMRI, which corresponded to the signs and symptoms of different CRPS phenotypes. Prior research has identified abnormalities in rsFC within multiple brain networks in patients with CRPS. These networks include the fronto-parietal [4,6], motor [3,7,10], somatosensory [5,6,11], subcortical [3,6,10,11], salience [4,8,9], central executive [4], and default mode [4,5] networks. These brain network alterations can be classified into two broad categories. The first category includes abnormalities in pain sensation [3-9,11] and cognitive-emotional changes [6,8]. The second involves limb movement disorders such as dystonia [3,4,10]. The father's diminished connectivity between the rostral PFC and OFC is associated with abnormalities in pain sensation and cognitive-emotional changes while the son's decreased connectivity between the globus pallidus and SMA is related to limb movement disorders such as dystonia.

Decrease in rostral PFC-OFC connectivity

Brodmann area 10, to which the rostral PFC belongs, plays a crucial role in the nociceptive process, including the integration and higher-order processing of nociception and pain [15]. This region is part of the prefrontal-parietal-thalamic circuit [15], and in patients with CRPS, a significant correlation has been identified between decreased rsFC within this subnetwork’s brain regions and deficits in cognitive-emotional pain processing [4,6]. Moreover, the OFC is involved in processing multifaceted external and internal sensory and emotional information and integrating cognitive functions through signal exchange with other regions, including the medial striatum, the mediodorsal thalamus, and additional prefrontal areas [16]. Considering the functions of these brain regions, the observed decrease in rsFC between the rostral PFC and OFC may be closely linked to the extensive sensory and cognitive impairments observed in the father.

Decreased pallidum-SMA connectivity

The SMA plays a critical role in motor control [17,18]. Motor commands from the SMA are transmitted to the putamen, a component of the striatum. The putamen then relays this information to another striatal component, the pallidum [19]. The pallidum modulates these signals and sends them back to the SMA via the thalamus [19]. Thus, the observed decrease in connectivity between the pallidum and SMA in the son may suggest a dysfunction in this feedback loop, potentially correlating with the patient’s dystonia and progressive musculoskeletal atrophy. In patients with CRPS, changes in connectivity within the basal ganglia motor loop, specifically between the basal ganglia and SMA, are associated with motor dysfunctions [3,10]. These findings demonstrate that the decreased pallidum-SMA connectivity noted in our patient may indicate abnormalities in the brain’s motor network.

Asymmetric rsFC changes

There may be a notable lateralization in the processing of pain information. In our case study, the father and son exhibited alterations in rsFC on the right side, which corresponds to the side of the affected limb. Current literature suggests that emotional and affective components of pain processing involve bilateral brain activity, often with a predominant involvement of the right hemisphere [20]. The changes in rsFC on the right side, noted in our patients, are in accordance with this perspective.

Limitations

Although abnormalities in rsFC appear to be closely associated with the phenotypes of our patients with CRPS, it is unclear whether these brain abnormalities are a consequence of peripheral sensory receptors or tissue and spinal cord abnormalities or whether the causal relationship is reversed. Additionally, we did not conduct specific genetic testing, which could shed light on genetic predispositions in our patients. Future research is imperative to investigate the causal links between rsFC abnormalities and peripheral tissue and spinal cord abnormalities in patients with CRPS. Additionally, genetic analyses should be performed. Such studies are crucial for understanding the pathophysiology and developing treatments for debilitating CRPS type 1.

## Conclusions

We have presented the case of a father and son diagnosed with CRPS type 1 who exhibited distinct aberrant rsFC patterns in fMRI analyses. These patterns correlated with their respective CRPS phenotypes. Our findings strongly support the hypothesis that variations in rsFC are closely linked to the phenotypes of CRPS type 1, suggesting that rsFC could serve as a potential biomarker for differentiating CRPS phenotypes. Further research is necessary to confirm the clinical significance of these rsFC aberrations in patients with CRPS type 1.
